# Migrated Hem-o-Lok clips in the neobladder with giant stone formation: a case report

**DOI:** 10.1186/s12894-023-01223-8

**Published:** 2023-03-30

**Authors:** Shang Xu, Xin-Ning Wang, Zi-Jie Wang, Tian-Wei Zhang, Yu-Hao Zhang, Yuan-Chao Cao, Wei Jiao

**Affiliations:** Department of Urology, The Affiliated Hospital of Qingdao University, Qingdao, 266000 Shandong Province China

**Keywords:** Bladder stone, Neobladder, Hem-o-Lok, Bladder migration, Case report

## Abstract

**Introduction:**

Neobladder urolithiasis is a rare but important delaying complication of orthotopic urinary diversion. We report a case of Hem-o-Lok (HOLC) migration into the neobladder with giant stone formation after orthotopic neobladder cystectomy.

**Case report:**

We report a case of a 57-year-old man with frequent urination and occasional discharge of stones 3 years after a laparoscopic orthotopic neobladder cystectomy. Computed tomography revealed a large round 3.5 cm calculus. An endoscopic neocystolitholapaxy was performed, and a Hem-o-Lok was found in the center of the stone.

**Conclusion:**

We described the case presentation, treatment and analysis of etiology of stone formation to avoid such complication.

## Introduction

Neobladder urolithiasis is a rare but important delayed complication of orthotopic urinary diversion after laparoscopic orthotopic neobladder cystectomy [1]. Stone formation can be caused by numerous factors including foreign matter, urinary infection, urinary stasis or retention, metabolic abnormalities related to intestinal urine diversion [2]. Since 1999, the HOLC is widely used in various laparoscopic operations due to its suture stabilization and easy application.[3] We describe a case of HOLC which migrated into the neobladder with stone formation 3 years after laparoscopic orthotopic neobladder cystectomy.

## Case report

**Fig. 1:**
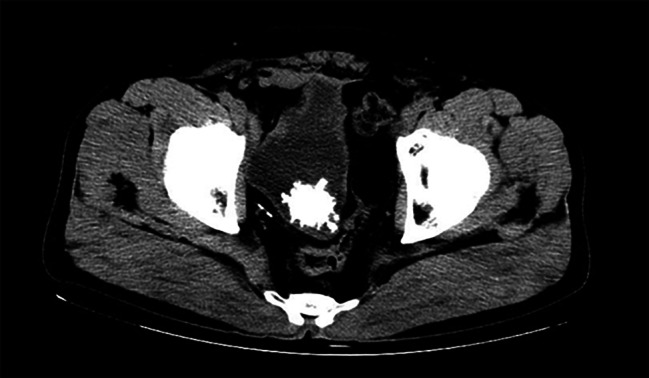
Cross view of computed tomography abdomen and pelvis shows large stone in the neobladder

**Fig. 2:**
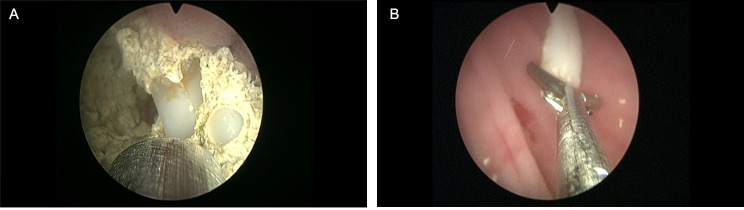
A Hem-o-Lok was encrusted by calculus (A); the intact Hem-o-Lok was removed by using forceps (B)

A 57-year-old man with frequent urination and occasional discharge of stones from the urethra was admitted to The Affiliated Hospital of Qingdao University. A laparoscopic orthotopic neobladder cystectomy was performed 3 years ago, and the post-surgery histopathology showed T2a muscle invasive bladder cancer. He did not continue the oncological follow-up. The patient complained of frequent urination and occasional stone discharge from the urethra. A urinary routine test showed urinary occult blood 2+, urinary leukocyte 1+, bacteria count 435.60/µl, and nitrite -. Computed tomography (CT) of the abdomen and pelvis revealed (Fig. 1): after total cystectomy, the ileum replaced the bladder, and a high-density shadow with a diameter of about 3.5 cm was observed inside. A diagnosis of “neobladder stone” was made. The patient had no history of underlying diseases, and his family had no history of urolithiasis. The patient underwent cystoscopy-assisted ultrasound lithotripsy immediately, a spherical large stone with rough surface and pus moss can be observed. When the stones were crushed, a closed white HOLC was found in the center of the stone (Fig. 2(A)). And the HOLC was removed with an endoscopic forceps (Fig. 2(B)). The infrared spectrum analysis of stone components indicated that ammonium magnesium phosphate, ammonium hydrogen urate, apatite carbonate. Six months after the removal of the whole stone, no voiding symptoms or local recurrence were found on the abdominal CT.

## Discussion

Laparoscopic orthotopic neobladder cystectomy is still the standard treatment for muscle invasive bladder cancer. In laparoscopic cystectomy, HOLC is used to ligate the vesical arteries, the vas deferens, the bilateral ureters, the seminal vesicle arteries, and the prostatic pedicles. Compared with titanium clips, HOLC have better histocompatibility and tolerance, and have less impact on CT and MRI examinations. However, due to its non-absorbability, HOLC occasionally cause tissue and organ perforation or migration. Shun-Fa et al. have reported a case of HOLC migrating into the bladder with stone formation after laparoscopic radical nephroureterectomy and bladder cuff excision [3]. Li Wan et al. also reported four cases of intrauterine device (IUD) migration into the bladder with stone formation [4]. After thoroughly searching the PubMed, there is only one report of Hem-o-Lok clip migration into the neobladder, which was reported by Shu-Xiong et al. As of yet, no definite mechanism has been identified for how surgical clips, including metal clips, migrate into the urinary tract. Shu-Xiong et al. hypothesized that the following contributes to HOLC migration: (1) the neobladder anastomosis represents a potential site for clip migration, (2) inflammation around the neobladder anastomosis may facilitate the HOLC eroding the anastomosis, (3) HOLC overlapped against the neobladder anastomosis may increase the pressure on the anastomosis, (4) eventually migration into the neobladder.

The patient was treated with the intracorporeal Studer technique, a neobladder reconstruction with folded detubularized ileu [5]. When dealing with prostatic apex, we use a hem-o-lok to clamp the lateral prostatic ligament, which is very close to the anastomosis of the neobladder to the posterior urethra. During cystoscopy, we found that the intestinal mucosal villi of the neobladder were atrophied, and no obvious scar was found on the wall of the neobladder, but the anastomotic scars was visible on the neck of the neobladder. We hypothesized that the hem-o-lok is likely to detach from the lateral prostatic ligament and migrate into the neobladder through this potential site in the anastomosis during long-term chronic inflammation.

Another concern is that HOLC migration into the neobladder can cause complications such as lower urinary tract symptoms, stone formation, or anastomotic fistula. Neobladder stones can be asymptomatic and discoverable incidentally on a radiological examination. However, with the enlargement of the stone, lower urinary tract symptoms are obvious. Tiu, A and Nguyen, S et al. respectively reported large neobladder stones of over 750 g [6, 7]. The main factor for the formation of this stone is chronic inflammation caused by foreign bodies in the neobladder, Kosan et al. found that it was the duration of time from having a foreign body in the bladder that was the greatest predictor of stone formation on suture materials [8]. In the presence of a urinary tract infection, urea is hydrolyzed in urine by urease-producing bacteria (Proteus, Klebsiella, pseudomonas and some Citrobacter species) to ammonia and carbon dioxide, which are further hydrolyzed to ammonium ions and bicarbonate. And then they combine with available cations produces magnesium ammonium phosphate and carbonate apatite, leading to the formation of infectious stones [9]. Another important factor is urinary retention, but the urinary control of the patient was well after surgery with only one pad a day and no urinary retention. Multiple factors such as neobladder mucus, and metabolic abnormalities associated with urinary intestinal diversion are also involved in the formation of neobladder stones [10]. The potential etiopathogenetic factors of the infectious stone formation are various, including the mucus production of bowel mucosa, the presence of urine infected by urease-producing germs, metabolic acidosis from ammonia absorption by urinary diversion, and increased urinary excretion of calcium and phosphate coupled with increased urinary pH and hypocitraturia, which can all contribute to increased risk of the infectious stone formation.

## Conclusion

Hem-o-Lok clip migration into the neobladder with stone formation after laparoscopic orthotopic cystectomy is an extremely rare complication that has been rarely reported in the literature. In order to prevent the migration of the HOLC and its complications, the use of the HOLC should be reduced under the premise of ensuring the effectiveness, and the loose HOLC should be removed in time. Regular re-examination of urine routine to avoid urinary infection and intermittent self-catheterization to prevent the occurrence of urinary retention may be effective methods to prevent the formation of neobladder stones.

## Data Availability

The de-identified data will be shared on reasonable request to the corresponding author.
